# Investigation by Imaging Mass Spectrometry of Biomarker Candidates for Aging in the Hair Cortex

**DOI:** 10.1371/journal.pone.0026721

**Published:** 2011-10-24

**Authors:** Michihiko Luca Waki, Kenji Onoue, Tsukasa Takahashi, Kensuke Goto, Yusuke Saito, Katsuaki Inami, Ippei Makita, Yurika Angata, Tomomi Suzuki, Mihi Yamashita, Narumi Sato, Saki Nakamura, Dai Yuki, Yuki Sugiura, Nobuhiro Zaima, Naoko Goto-Inoue, Takahiro Hayasaka, Yutaka Shimomura, Mitsutoshi Setou

**Affiliations:** 1 Department of Cell Biology and Anatomy, Hamamatsu University School of Medicine, Hamamatsu, Shizuoka, Japan; 2 Laboratory of Genetic Skin Diseases, Niigata University Graduate School of Medical and Dental Sciences, Niigata, Niigata, Japan; University Hospital Hamburg-Eppendorf, Germany

## Abstract

**Background:**

Human hair is one of the essential components that define appearance and is a useful source of samples for non-invasive biomonitoring. We describe a novel application of imaging mass spectrometry (IMS) of hair biomolecules for advanced molecular characterization and a better understanding of hair aging. As a cosmetic and biomedical application, molecules whose levels in hair altered with aging were comprehensively investigated.

**Methods:**

Human hair was collected from 15 young (20±5 years old) and 15 older (50±5 years old) volunteers. Matrix-free laser desorption/ionization IMS was used to visualize molecular distribution in the hair sections. Hair-specific ions displaying a significant difference in the intensities between the 2 age groups were extracted as candidate markers for aging. Tissue localization of the molecules and alterations in their levels in the cortex and medulla in the young and old groups were determined.

**Results:**

Among the 31 molecules detected specifically in hair sections, 2—one at *m/z* 153.00, tentatively assigned to be dihydrouracil, and the other at *m/z* 207.04, identified to be 3,4-dihydroxymandelic acid (DHMA)—exhibited a higher signal intensity in the young group than in the old, and 1 molecule at *m/z* 164.00, presumed to be *O*-phosphoethanolamine, displayed a higher intensity in the old group. Among the 3, putative *O*-phosphoethanolamine showed a cortex-specific distribution. The 3 molecules in cortex presented the same pattern of alteration in signal intensity with aging, whereas those in medulla did not exhibit significant alteration.

**Conclusion:**

Three molecules whose levels in hair altered with age were extracted. While they are all possible markers for aging, putative dihydrouracil and DHMA, are also suspected to play a role in maintaining hair properties and could be targets for cosmetic supplementation. Mapping of ion localization in hair by IMS is a powerful method to extract biomolecules in specified regions and determine their tissue distribution.

## Introduction

### Roles of human hair

Hair significantly influences the appearance and is one of the components of the human body that determine how individuals look for their age [1]. Hair changes chemically and physically as a result of various environmental assaults and undergoes intrinsic degeneration with aging, resulting in an alteration of its appearance, *e.g.*, color and shine; feel, *e.g.*, wettability and softness; and structure, *e.g.*, formation of split ends and frizz [2]. Therefore, hair care is a huge industry, which supplies products such as shampoos and conditioners to clean, protect, and provide a desirable look and feel to hair [3].

At the same time, hair is used as an index of body properties. The advantages of hair over other commonly used samples such as blood or urine as an indicator include ease and painlessness of sampling, ease of storage, and the possibility of monitoring past exposure [4], [5], [6]. Forensically, hair has been utilized as trace evidence for the investigation and successful prosecution of individuals suspected of being involved in crimes [7].

### Molecules constructing human hairs

Hair keratin proteins and hair keratin-associated proteins (KAPs), composed of large gene families, are the predominant structure proteins in the hair [8], [9]. In the human hair cortex, keratin intermediate filaments (KIFs) are produced from hair keratins, cross-linked with KAPs through extensive disulfide bonds, and rigid hair shafts are produced.

### Methods for molecular research of hair

Nearly all methods reported in the literature identify analytes in the hair by headspace solid phase microextraction-gas chromatography-mass spectrometry (MS), or more recently, by gas chromatography-tandem MS [10] and liquid chromatography-MS [11]. However, as samples are generally prepared by the elution of molecules with an organic solvent, these methods provide neither visual information nor analyte localization within the hair strand. In this regard, several techniques involving secondary ion MS and multi-isotope imaging MS (IMS) are used for the determination of elemental composition of a cross-section of hair; however, detectable targets are limited to elements [12], [13], [14].

Matrix-assisted laser desorption/ionization (LDI) or LDI-based IMS enables the analysis of much larger biomolecules because of the soft ionization principle used and is a powerful tool for investigating biomolecules comprehensively without the use of time-consuming extraction, purification, or separation procedures for biological tissue sections [15], [16], [17]. Although IMS is used for detecting drugs in the hair for forensic purpose [18], comprehensive analysis of molecules in hair by using IMS is yet to be done. Earlier, we developed an imaging mass spectrometer with a higher spatial resolution than the original ones [19]. This was utilized for IMS analysis of hair sections in the present study.

### Molecular markers for aging in hair

The characterization of hair aging with regard to the alteration of molecular mass and distribution of molecules within the hair structure is essential for the development of better cosmetic products. It is predicted that in the future, improvements in hair-care product development will target specific molecules [20], and thus, the supplementation of molecules impaired during aging or addition of their functional analogues to hair care products would be beneficial to this section of the market.

The identification of molecules in hair that could define an individual's age has important forensic applications. This is because the age, which is one of most vital pieces of information in an investigation, cannot be determined definitively by the use of microscopically measured indices such as hair diameter or ellipticity [7], [21].

Molecules related to aging in hair, however, have not been comprehensively investigated, with the exception of some trace minerals analyzed by atomic absorption spectrometry [22], [23]. Therefore, as the first target for utilizing our method, we chose investigation of biomarkers that could distinguish human age.

The objective of this article is to describe an initial assessment of the application of IMS for comprehensive detection of aging-related molecules in cross-sectioned hair.

## Results

### Visualization of molecular distribution in cross-sectioned hair

**Figure 1:**
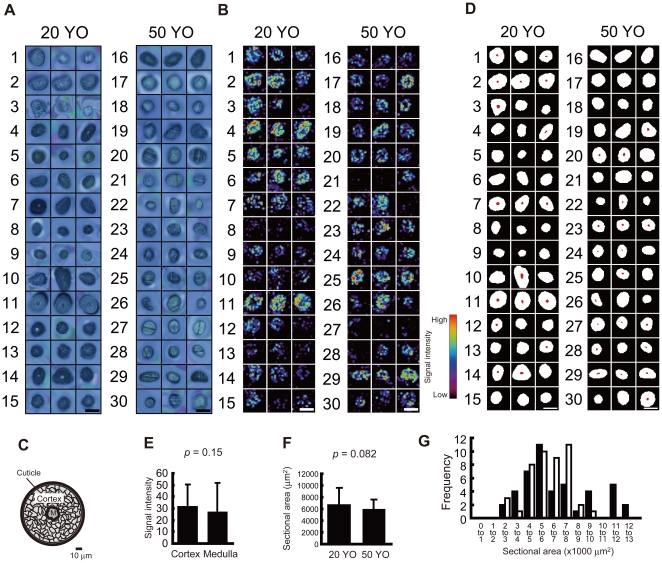
Visualization of molecular distribution in hair cross-sections. (A) Images of cross-sectioned hairs are shown, numbered as per the subject No. Each of the 3 photographs with the same number is from an independent scalp hair. Scale bar: 100 µm. (B) Ion distribution at *m/z* 125.99. Scale bar: 100 µm. (C) The principal hair structures are shown. M: hair medulla. (D) ROI corresponding to the hair structures are depicted. White area: cortex. Red area: medulla. Black area: background. Scale bar: 100 µm. (E) The signal intensity at *m/z* 125.99 in hair cortex and medulla is shown *: *p*<0.05. (F) Cross-sectional area of hair is shown. (G) A histogram of the cross-sectional area is depicted. Black bar: 20-YO group. White bar: 50-YO group. All values are presented as mean±standard deviation.

Figure 1A provides images of hair sections obtained from subjects aged 20±5 years (hereafter termed 20 YO group) and 50±5 years (50 YO group). As a typical result of IMS analysis, the ion distribution at a mass-to-charge ratio (*m/z*) of 125.99, detected with the highest intensity among hair-specific ions, is presented in Figure 1B.

Human scalp hair is composed of a core structure of a centrally located medulla, cortex consisting of different cell types, and surrounding layer of cuticle cells (Figure 1C)[24]. Figure 1D depicts the definition of regions of interest (ROI) corresponding to cortex and medulla. As shown in Figure 1A, S1, and S2, the shape and size of hair sections were different among samples. Thus, the areas that were considered to correspond to the medulla were observed only in 38 hair sections out of 90 in the light microscopic images of the sections. Mean signal intensity of the ion at *m/z* 125.99 in the cortex did not show statistically significant difference from that in the medulla (*p* = 0.15, paired *t*-test; Figure 1E).

In order to confirm that cross-sectional area does not show linear alteration with aging and thus cannot be used as an indicator of aging, the area composed of both the cortex and medulla was counted and compared between the 2 age groups. There was no significant difference in cross-sectional area between the 20 YO and 50 YO group (*p* = 0.082, unpaired *t*-test; Figure 1F), while large sectional area over 11,000 µm^2^ was only seen in the 20-YO group (Figure 1G).

### Selection of hair-specific molecules

As a preliminary step in the detection of markers for aging, principal hair-specific molecules were extracted. The top 50 signals in the ROI in the cortex and medulla were selected from each of signal intensity values obtained from the analysis of subjects No. 1, 2, 3, 16, 17, and 18 (Figure S3A), and 56 ions chosen from more than 1 of these 6 subjects were listed (Figure S3B). Among these, 31 ions with a mean hair-specific intensity at least 3-fold and statistically significantly (*p*<0.05, paired *t*-test) higher than the mean background intensity (black area in Figure 1D) were selected as hair-specific molecules (red letters in Figure S3B).

### Extraction of putative aging markers

**Figure 2:**
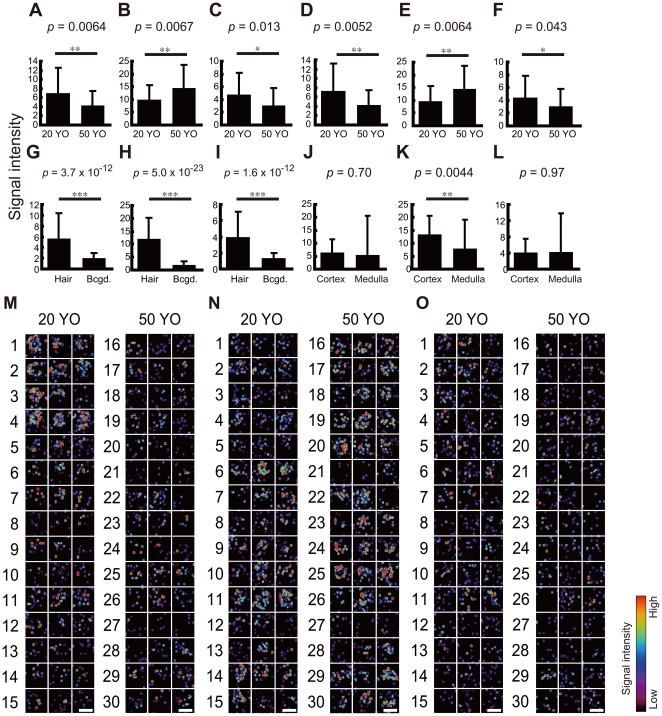
Intensity and distribution of putative aging markers. (A–C) Mean signal intensity in the hair section is displayed. (D–F) Mean signal intensity in the hair section excluding those with sectional area of 11,000 mm^2^ or larger is displayed. (G–I) Mean signal intensity in the hair section and background area is displayed. (J–L) Mean signal intensity in the cortex and medulla is displayed. (M–O) Each panel shows ion distribution in cross-sectioned hair. The number indicates the subject No. Each of the 3 pictures was obtained from an independent scalp hair of a single subject. Scale bar: 100 µm. (A, D, G, J, M) *m/z* 153.00. (B, E, H, K, N) *m/z* 164.00. (C, F, I, L, O) *m/z* 207.04. All values are shown as mean±standard deviation. *: *p*<0.05. **: *p*<0.01. ***: *p*<10^−12^.

In order to find putative aging markers, those molecules that exhibited a significant difference in the signal intensity between the 2 age groups from among the 31 hair-specific molecules were determined. The mean signal intensities at *m/z* 153.00 and 207.04 were higher in the 20 YO group than in the 50 YO group (*p* = 0.0064 and 0.013, respectively; unpaired *t*-test; Figures 2A and C). In contrast, the ion at *m/z* 164.00 was observed to have a significantly higher intensity in the 50 YO group than in the 20 YO group (*p* = 0.0067; Figure 2B). Even when the sections whose areas were over 11,000 µm^2^ were excluded from the analysis to reduce possible diameter effects, the significance of the differences was not altered (*p* = 0.0052, 0.0064, and 0.043 for *m/z* 153.00, 164.00, and 207.04, respectively; unpaired *t*-test; Figure 2D, E, and F).

As already evaluated by selection of hair-specific ions, these 3 molecules were specific to hair sections (Figure 2G, H, and I **)**. Among the 3 putative aging markers, only the one at *m/z* 164.00 presented a cortex-specific distribution (*p* = 0.70, 0.0044, and 0.97 for *m/z* 153.00, 164.00, and 207.04, respectively; paired *t*-test; Figure 2J, K, and L). Hair-specific distribution of the molecules and cortex-specific distribution of the molecule at *m/z* 164.00 in each subject's sample was confirmed by mapping of ion distribution (Figure 2M, N, and O).

**Figure 3:**
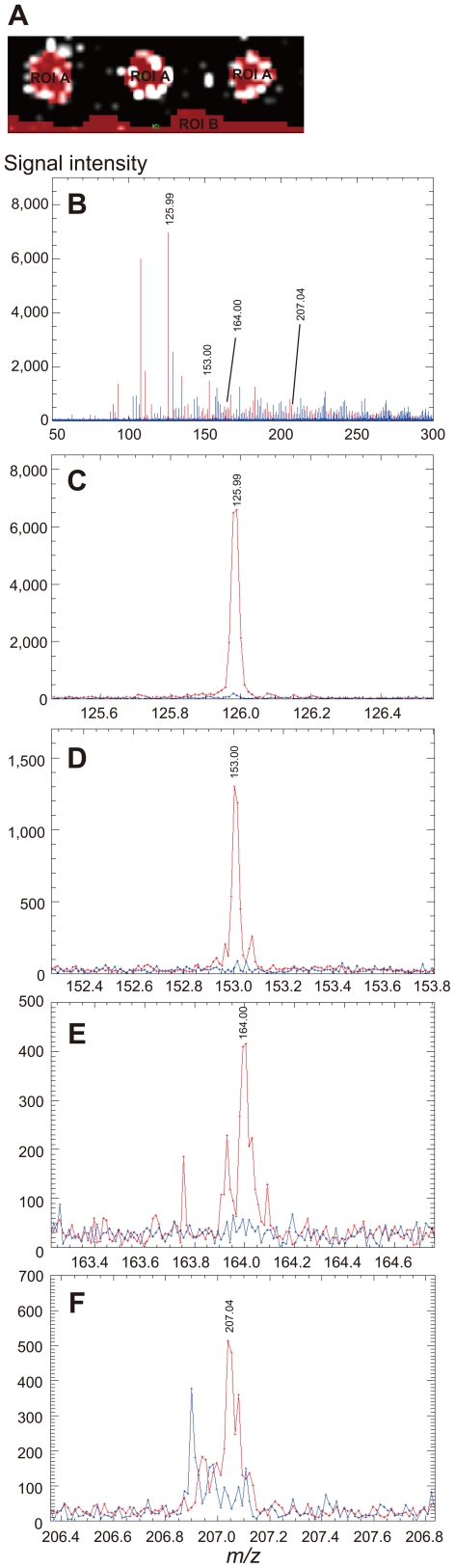
Hair-specific mass spectra of putative aging markers. ROI-specific mass spectra in subject No. 1 are presented. Red peaks and blue peaks are derived from the hair section and background area, respectively. (A) ROI selection is illustrated: ROI A as hair section and ROI B as background area. (B) *m/z* 50 to 300. (C) *m/z* 125.99. (D) *m/z* 153.00. (E) *m/z* 164.00. (F) *m/z* 207.04.

To confirm that the extracted molecule originated from a single molecule observed as a peak in the histogram, ROI-specific mass spectra were structured using values from subject No. 1 as a typical example. As illustrated in Figures 3B to F, signal intensity at *m/z* 125.99, 153.00, 164.00, and 207.04 in hair-specific ROI (red color) were observed as individual peaks.

### Comparison of intensity of the putative markers in the cortex and medulla between 2 age groups

**Figure 4:**
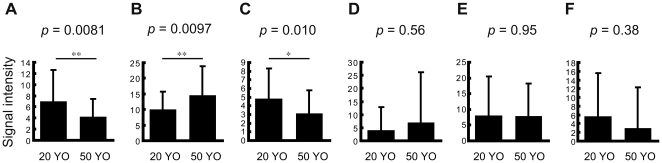
Putative aging marker intensity in the cortex and medulla. (A–C) Mean signal intensity in the hair cortex in the 20 YO and 50 YO groups is displayed. (D–F) Mean signal intensity in the hair medulla in the 20 YO and 50 YO groups is displayed. (A, D) *m/z* 153.00. (B, E) *m/z* 164.00. (C, F) *m/z* 207.04. All values are shown as mean±standard deviation *: *p*<0.05.

To determine whether the intensity of the molecule in the cortex and medulla differed between 20 YO and 50 YO, mean intensity in each ROI was compared between the 2 age groups. Figures 4A and C illustrate that the intensities at *m/z* 153.00 and 207.04 in the cortex were significantly higher in the 20 YO than in the 50 YO group (*p* = 0.0081 and 0.010, respectively; unpaired *t*-test). As shown in Figure 4B, the ion at *m/z* 164.00 in the cortex displayed a higher intensity in the 50 YO than in the 20 YO (*p* = 0.0097). On the other hand, no significant difference between the 2 groups was observed in the mean medullary intensity at any *m/z* value (*p* = 0.56, 0.95, and 0.38 for *m/z* 153.00, 164.00, and 207.04, respectively; Figure 4D, E, and F).

### Assignment of putative aging markers

Table 1 shows the assignment of the putative aging markers extracted above, listing the molecules which exhibited highest probability in MS/MS analysis and the molecules assumed in single MS analysis.

**Table 1:** Assignment of molecules at *m/z* 125.99, 153.00, 164.00, and 207.04 (* indicates the molecule identified by MS/MS analysis.).

Measured *m/z*	Compound ID	CAS No.	Compound name	Chemical formula	Molecular weight	Adduct	Culculated adduct *m/z*
125.99	METLIN58164	1948-56-7	2-Aminoacrylic acid	C_3_H_5_NO_2_	87.03	[M+K]^+^	126.00
153.00	HMDB00076	504-07-4	Dihydrouracil	C_4_H_6_N_2_O_2_	114.04	[M+Na]^+^	153.01
164.00	HMDB00224	1071-23-4	*O*-Phosphoethanolamine	C_2_H_8_NO_4_P	141.02	[M+Na]^+^	164.01
207.04	HMDB01866	775-01-9	3,4-Dihydroxymandelic acid*	C_8_H_8_O_5_	206.33	[M+Na]^+^	207.33

From the precursor ion at *m/z* 207.04, fragment ions at *m/z* 75.49, 133.07, 138.89, 141.84, 149.53, 151.51, 188.32, and 198.92, fragment ions at *m/z* 140.88 and 160.26, fragment ions at *m/z* 61.06, 84.82, 136.18, 143.88, 148.54, and 191.53 were detected at the energy level of 10, 30, and 50, respectively (Figure S4A to E). By searching the fragments obtained at energy level of 10, 30, and 50, the precursor ion was assigned to 3,4-dihydroxymandelic acid (DHMA), which presented the highest relative fitness values among all precursor candidates: 81%, 98%, and 96%, respectively.

*M/z* 153.00 and 164.00 were speculated to be dihydrouracil and *O*-phosphoethanolamine, respectively, which showed a difference of 0.01 Da or less between the calculated *m/z* and query. The ion with the highest intensity among hair-specific ions, *m/z* 125.99, was assigned to 2-aminoacrylic acid.

## Discussion

In the present study, we applied IMS to cross-sectioned hair to investigate multiple molecules showing aging-related alterations. Images of hair sections were obtained at high resolution, and the distribution of ions was characterized on a micrometer scale.

### Speculated relationship between aging and tissue localization of the extracted molecules

Dihydrouracil, presumed to be a precursor of the ion at *m/z* 153.00, is an intermediate metabolite of uracil [25], and DHMA, identified as the ion at *m/z* 207.04, is a major metabolite of the catecholamines [26]. Both are released into the circulation after conversion [25], [26] and can thus be transferred to matrix cells and/or melanocytes via blood vessels in the dermal papilla [27].

Increased apoptosis of follicular melanocytes is a phenomenon associated with aging [28]. Eumelanin synthesized in bulbar melanocytes is transferred to matrix cells which proliferate and differentiate into the hair shaft cortex [29]. The robust binding of eumelanin to basic molecules by ionic interaction [30] and aromatic carbon by hydrophobic interaction [31] renders it a drug-binding site within the hair structure, therefore, the hydrophobic base dihydrouracil and DHMA with dihydroxybenzene ring are foreseeable interaction partners. This might explain the relationship between aging and reduction of the signal intensities, and further investigation would be done to validate this theory.

Dihydrouracil and DHMA were detected in a punctuated, heterogenous manner through the hair sections. This heterogeneity might be explained by the diversity of cellular components of the hair structure, since a hair fiber is composed of different cell types including the ortho-, the meso-, and the paracortical cells [32]. Moreover, recent study has shown that KIFs in the fiber has several patterns in their arrangement [24]. If such structural molecules in the hair shaft function as an absorber for the two molecules or their precursor molecules, the heterogeneity which was observed here will result.

*O*-phosphoethanolamine, tentatively assigned to the ion at *m/z* 164.00, is a metabolite of sphingosine-1-phosphate (S1P) [33]. Aging is associated with increased platelet activation [34], which leads to enhanced secretion of S1P into the circulation [35]. As observed in other tissues, increased S1P might be processed to *O*-phosphoethanolamine and 2-hexadecanal by S1P-lyase following internalization by the cells forming the hair structure [36].

The mean signal intensity at *m/z* 125.99, tentatively assigned to 2-aminoacrylic acid, did not significantly differ between that in the hair cortex from in the medulla (Figure 1E). Both cortical and medullar cells are composed mostly of bundles of KIFs and consist of keratin proteins [24], [37], while the hair medulla contains heavily vacuolated cells. Since 2-aminoacrylic acid, also called dehydroalanine, is a product of post-translational modifications observed in keratin proteins, its localization both in the cortex and the medulla is reasonable [38].

### Cosmetic perspective

The hair shaft surface is covered with integral lipids, the only continuous structure that plays a role in maintaining moisture, luster, mechanical integrity, and stiffness of hair [2], [39]. Oil-containing hair cosmetics have been experimentally proven to complement or raise the efficacy of endogenous lipids: coconut oil to prevent moisture diffusion from the hair [40] and castor oil to increase luster [41]. Artificial compounds are also utilized to imitate the function of this integral lipid such as modified silicone oils to maintain luster [41] and prevent breakage [42].

As dihydrouracil and DHMA have a hydrophobic structure due to pyrimidinedione and benzenediol, these molecules will have roles in the integral lipids. Since these levels are impaired with aging, their supplementation or the addition of mimic molecules might be effective in improving the function of cosmetic products. Moreover, since KAPs are hydrophobic and predicted to attach to these molecules [8], these might directly bind to KIFs, and increase hair rigidity and appearance.

### Endogenicity of extracted molecules

Certain hair products such as shampoo, conditioner, and hair dye contain compounds classified as having the same organic structure as the candidate markers we identified. For instance, among the ethanolamines, diethanolamine is utilized for forming [43]. However, the possibility that the 3 molecules are derived from such hair products is limited, because they are not registered in the 2 comprehensive lists of ingredients used in cosmetic products, compiled by the American Cosmetic Association [44], [45] and the Japan Cosmetic Industry Association [46].

### Principles for further investigation

Identification and confirmation of the functional properties of molecules discussed above would be pursued in the course of further studies. For the functional characterization of the cosmetic applications of dihydrouracil and DHMA, their effect on hair with regard to molecular aspects such as composition of integral lipids and moisture deposition, morphological aspects such as luster and smoothness, and mechanical aspects such as softness and stiffness merit investigation. For forensic purposes, the applicability of the 3 molecules for identifying individual age will be evaluated.

Hair analysis with this technique would be repeated to clarify some points raised from this study. For instance, the molecular mechanism of aging-related reduction of dihydrouracil and DHMA in cortical area, which we proposed is due to their binding properties to melanine, remains to be investigated in both white and black hairs. While we defined the medulla from the optical microscopic images in the present study, molecular definition of the medulla using specific markers would be effective for more accurate analysis. Moreover, analysis of hair sections from the root to the top would be significant to clarify three-dimentional distribution of the extracted molecules.

Since, in our study, the analyte was ionized in a matrix-free condition, matrix-assisted LDI might lead to the discovery of other molecular marker candidates. In addition, recent techniques have enabled the application of various types of matrices to IMS analysis and to the optimization of matrix composition [47], [48], [49]. Application of novel matrices such as a nano-particle matrix helps detect novel molecular species [47], [50].

Sample availability restricted our analysis to hair from Japanese female donors. Analyzing male hair or hairs from different ethnic origins would be a step towards further investigation of the present results. As a procedure to distinguish sexes based on hair has not been established [7], investigation of molecules with differential intensities or different distribution between sexes could be significant.

Importantly, this technique enabled simultaneous imaging of fine optical feature and molecular distribution in a single hair section, which allowed analysis of ion distribution and discrimination of hair structures, such as cortex and medulla, in a single sample. The technique, therefore, would be beneficial in every field focusing on the molecular distribution in the hair regarding tissue structure.

In conclusion, we succeeded in applying newly developed Mass Microscopy to cross-sectioned hair, visualizing molecular distribution in the hair sections. As an initial target for the application of hair IMS, molecules showing a change in the ion intensity with aging were comprehensively investigated, and 3 molecules with altering levels in the cortex were found. They were strong candidates for aging markers, and 2 of these molecules, dihydrouracil and DHMA, are proposed as the cosmetic target molecules.

## Materials and Methods

### Ethics statement

All experiments in this study were specifically approved by the Ethics Committee at the Hamamatsu University School of Medicine. Subjects consented in written form to cooperate after they were informed that they would not incur any disadvantage, that they could resign from the study, that the researchers were obliged to protect privileged information, that any identity will not be revealed, and that the obtained samples will be eradicated after the study. During sampling, care was taken to preserve the subjects' appearance and to ensure that the subjects were not distressed.

### Reagents

Carboxy methyl cellulose was obtained from Wako Pure Industries Ltd, Japan (Osaka, Japan). Indium-tin-oxide-coated slide glasses were obtained from Bruker Daltonics (Bremen, Germany).

### Subjects

This study was performed between August and December 2010 in healthy female Japanese adults divided into 2 groups of those aged 20±5 years and those aged 50±5 years.

### Sample preparation

Three strands of hair per subject were collected in order to minimize the effect of scalp location. Hair from the parietal region was cut at 1-cm distance from the skin. A 1-cm-long section from the root side of the hair was cut and immersed in 2% carboxy methyl cellulose, rapidly frozen in liquid nitrogen, and 8-µm-thick sections were cut perpendicular to the longitudinal axis in a CM1950 cryostat (Leica Microsystems, Wetzlar, Germany) at −20°C. The section was mounted on a glass slide coated with indium-tin-oxide.

### Imaging mass spectrometry analysis

All IMS experiments were performed in the positive ionization mode using MS-IT-TOF (Mass microscope; Shimadzu Corporation, Kyoto, Japan) in the linear positive mode. Nd:YAG laser at 355 nm was used at 40% energy (8 µJ/pulse) and 1000 Hz repetition rate. The interval between data points was 10 µm, yielding a total of 289 data points, sufficient to cover the entire section. Mass spectra were obtained with a scanning mass range of 50 to 300 Da with the mass resolution of 10,000. Images of hair sections were acquired using the Mass microscope prior to LDI.

### MS/MS analysis

MS/MS experiments on hair section were performed by using scalp hairs of a healthy 31-years-old Japanese subject. In order to obtain sufficient signals, one hair was used for each measurement. The molecular weight range for the ion trapping was 1.0 Da around *m/z* of each precursor ion. The setting of IMS analysis was as follows: The laser intensity for fragmentation of either 0, 10, 30, or 50; gas level of 50; accumulating time of 221 msec; repeating number of 1; interval between data points of 5 µm. The other setting status was the same as that of MS analysis of precursor ions.

### Comparison of signal intensities in the cortex and medulla

All IMS data were integrated and normalized with total ion currents by SIMtools software (in-house software; Shimadzu Corporation) and imported to Biomap Ion Imaging Software ver. 3.7 (Novartis Institutes for BioMedical Research, Basel, Switzerland). TIFF images of specified *m/z* were generated and imported to ImageJ software ver. 1.4 (National Institutes of Health, Bethesda, MD). ROI in the hair section of each subject were defined by tracing an outline on the image. The hair sections with the central area with the brightness different from that of the peripheral area were considered to have medullary structure. ROI of the medulla was defined as that area, and ROI of the cortex, as the rest of the section. Only the sections whose medullary areas were observed were used for statistical analysis for comparing signal intensity in cortex and medulla. The mean graphical intensity of each subject's ROI was measured by ROI analysis function and exported as a Microsoft Office Excel file. Mean and standard deviation values of the graphical intensities were calculated by Microsoft Office Excel 2007 software (Microsoft Corporation, Redmond, WA). Difference of mean intensity in the cortex and medulla was assessed using paired Student's *t*-test, and a *p* value of <0.05 was considered significant.

### Comparison of sectional area between age groups

Total areas of the ROI measured in ImageJ were compared using unpaired Student's *t*-test, and a *p* value <0.05 was considered significant. The sectional area of each hair was classified into segments from 0 to 13,000 µm^2^ and the frequency of the number of the hairs in each segment was depicted as a histogram.

### Selection of hair-specific molecules

Signal intensity values obtained by IMS analysis in subject Nos. 1, 2, 3, 16, 17, and 18 were imported into SIMtools. The peak-picking procedure was performed to select the top 50 ions in the range of *m/z* 50 to 300 with a molecular weight tolerance of 0.05 Da in the ROI of the section. Distribution of an ion selected in more than 1 of these subjects was visualized by Biomap as an integrated image that included results from all subjects, and exported to ImageJ. The area other than the hair section was defined as background ROI. Mean graphical intensities in ROI corresponding to each subject's hair and background area were measured. Hair-specific ions were defined as ions that met both of following conditions: (1) The mean intensity in the hair was 3-fold or more of that in the background. This is a typical method of peak selection in IMS, considering a peak with a signal:noise ratio greater than 3:1 to be significant [51]. (2) Difference between mean intensity in hair and that in the background was significant by paired Student's *t*-test, with a *p* value of <0.05.

### Comparison of signal intensities between age groups

A mean graphical intensity in the ROI corresponding to each subject's hair, cortex, or medulla, was measured. Difference between mean intensities in the 2 age groups was assessed by unpaired Student's *t*-test, and a *p* value of <0.05 was considered significant.

To reduce the influence of the diversity in sectional areas to the result, the analysis excluding hairs with extra-larger sectional areas was also performed. The first and second sections of Subject No.2, the second section of Subject No.10, all three sections of Subject No.11, and the second section of Subject No.14 were excluded.

### Region-of-interest-specific mass spectra

IMS results obtained from 3 hair sections of subject No. 1 were integrated and normalized to total ion currents using SIMtools and then exported to Biomap. Mass spectra in the ROI corresponding to the hair and in the background area were depicted by the ROI Plot procedure.

### Assignment of molecules

A search for candidate molecules corresponding to the ion precursors of the rest of the extracted ions was performed in Human Metabolome Database (HMDB; Genome Alberta, Alberta, Canada; http://www.hmdb.ca/) and The METLIN Metabolite Database (Scripps Center for Metabolomics, La Jolla, CA; http://metlin.scripps.edu/). Molecules with *m/z* differences from the calculated values less than 0.01 Da were searched for. Since alkali metal adduct ions predominate in the positive ion mode of LDI [52], a molecule adducted with a sodium ion ([M+Na]^+^) and another with a potassium ion ([M+K]^+^) were selected as candidates. Drug metabolites and molecules from non-human organisms were excluded from the lists.

Assignment of the precursor molecule by tandem mass analysis was performed by using MS/MS Search section of HMDB. Peaks with signal intensity which exceeded the baseline signal 2-fold or higher were considered as major fragments obtained in secondary ionization of precursor molecules. The values of *m/z* and the relative intensities of these peaks were assigned as queries, and a molecule with the highest relative fitness values were searched. Fragments from the precursor ion at *m/z* 207.04 at energy level of 0, 10, 30, and 50 in Mass Microscope were regarded to correspond to data at “Low”, “Middle”, and “High” Energy Level in HMDB, respectively.

## Supporting Information

Figure S1 **Light microscopic images of hair sections of 20-YO group.** High resolution microscopic images of the hair sections from the subjects of 20-YO group are presented. *: The section in which medulla was defined. Scale bar: 100 µm.
(TIF)

Figure S2 **Light microscopic images of hair sections of 50-YO group.** High resolution microscopic images of the hair sections from the subjects of 50-YO group are presented. *: The section in which medulla was defined. Scale bar: 100 µm.
(TIF)

Figure S3 **Extraction of hair-specific molecules.** (A) The *m/z* values with the top 50 intensities in the hair-specific ROI of each subject are listed. The number over the image indicates the subject No. Solid lines in a visual image indicate ROI. Scale bar: 100 µm. Bold letter: A value selected in more than 1 subject. (B) *M/z* values selected in more than 1 subject in (A) are listed. Red letter: Finally selected hair-specific molecule.
(TIF)

Figure S4 **MS/MS analysis.** (A–D) Mass spectra of secondary ions by fragmentation of the precursor ion at *m/z* 207.04 are presented. Measurement of precursor ions at energy level of 0. (B) Fragmentation at energy level of 10. (C) Fragmentation at energy level of 30. (D) Fragmentation at energy level of 50. The inserted pictures present the area on the hair sections on which the lasor was pulsed for ionization. (E) Light microscopic images of the hair sections are shown. Lasor-pulsed areas are framed. Scale bar: 50 µm.
(TIF)
