# Supplementary figures and images for: Investigation by Imaging Mass Spectrometry of Biomarker Candidates for Aging in the Hair Cortex
Source: PLoS One. 2011 Oct 24;6(10):e26721. doi: 10.1371/journal.pone.0026721 (PMC3200353; doi:10.1371/journal.pone.0026721)

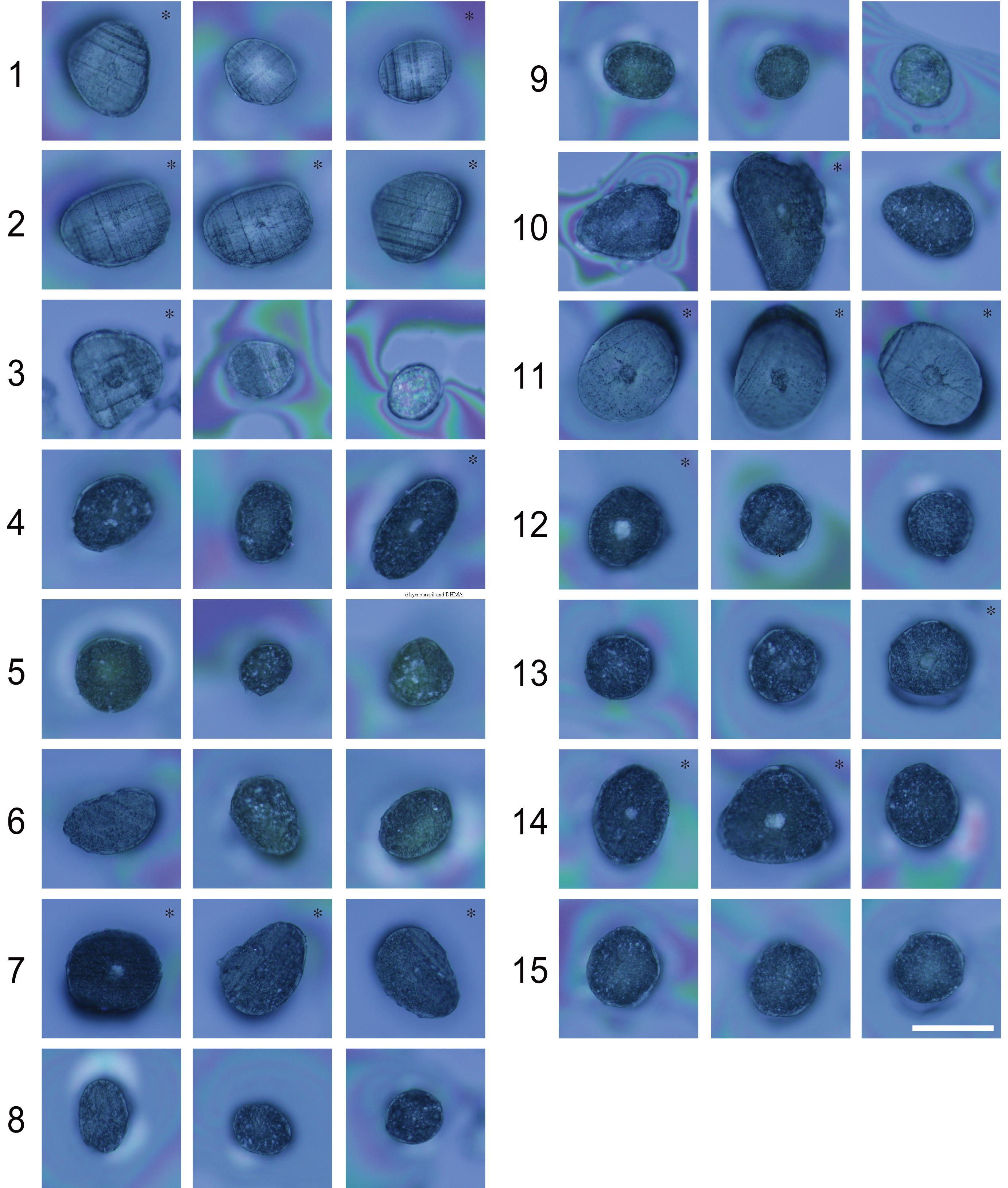

Supplement: Figure S1 — Light microscopic images of hair sections of 20-YO group. High resolution microscopic images of the hair sections from the subjects of 20-YO group are presented. *: The section in which medulla was defined. Scale bar: 100 µm. (TIF) [file pone.0026721.s001.tif]

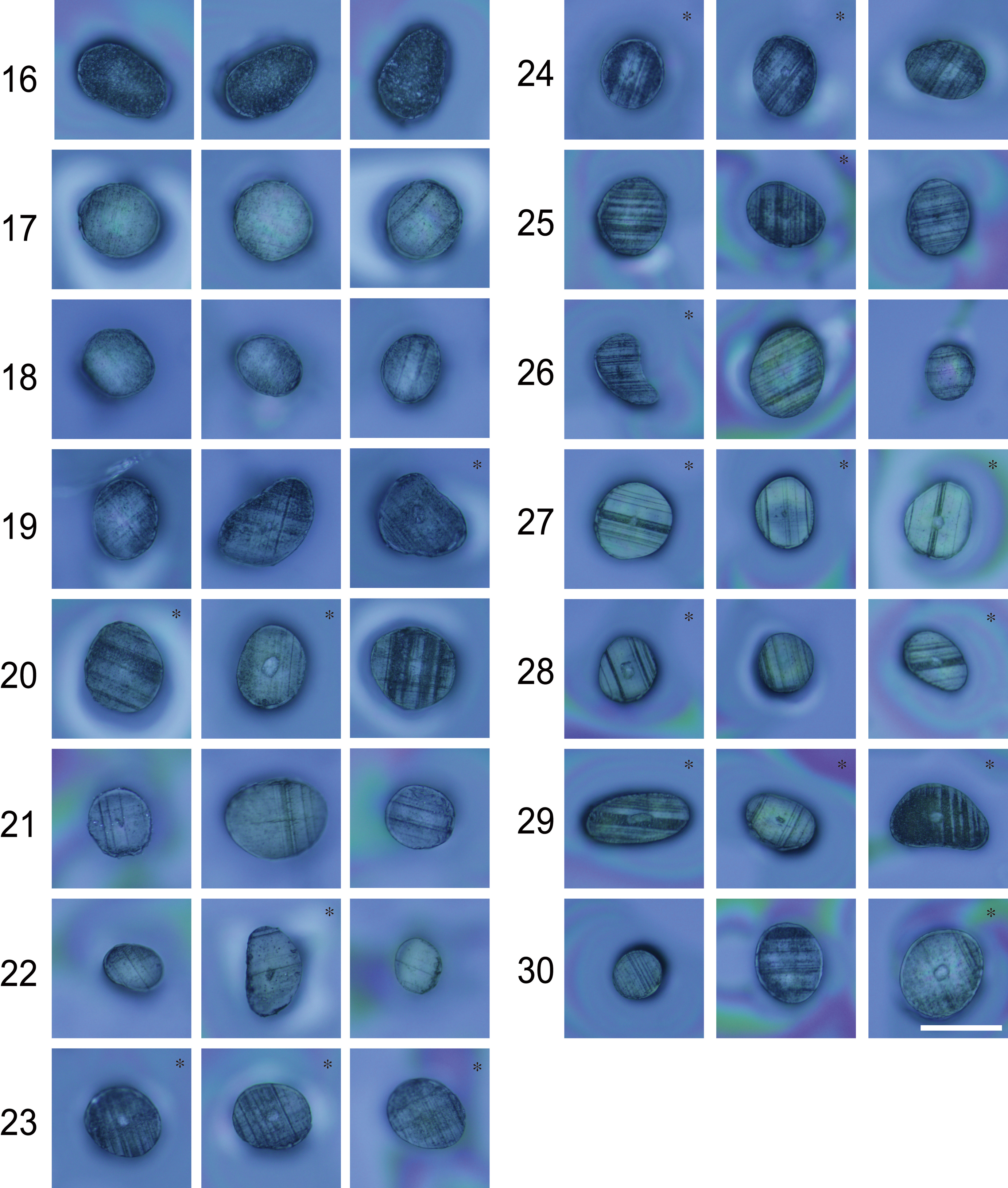

Supplement: Figure S2 — Light microscopic images of hair sections of 50-YO group. High resolution microscopic images of the hair sections from the subjects of 50-YO group are presented. *: The section in which medulla was defined. Scale bar: 100 µm. (TIF) [file pone.0026721.s002.tif]

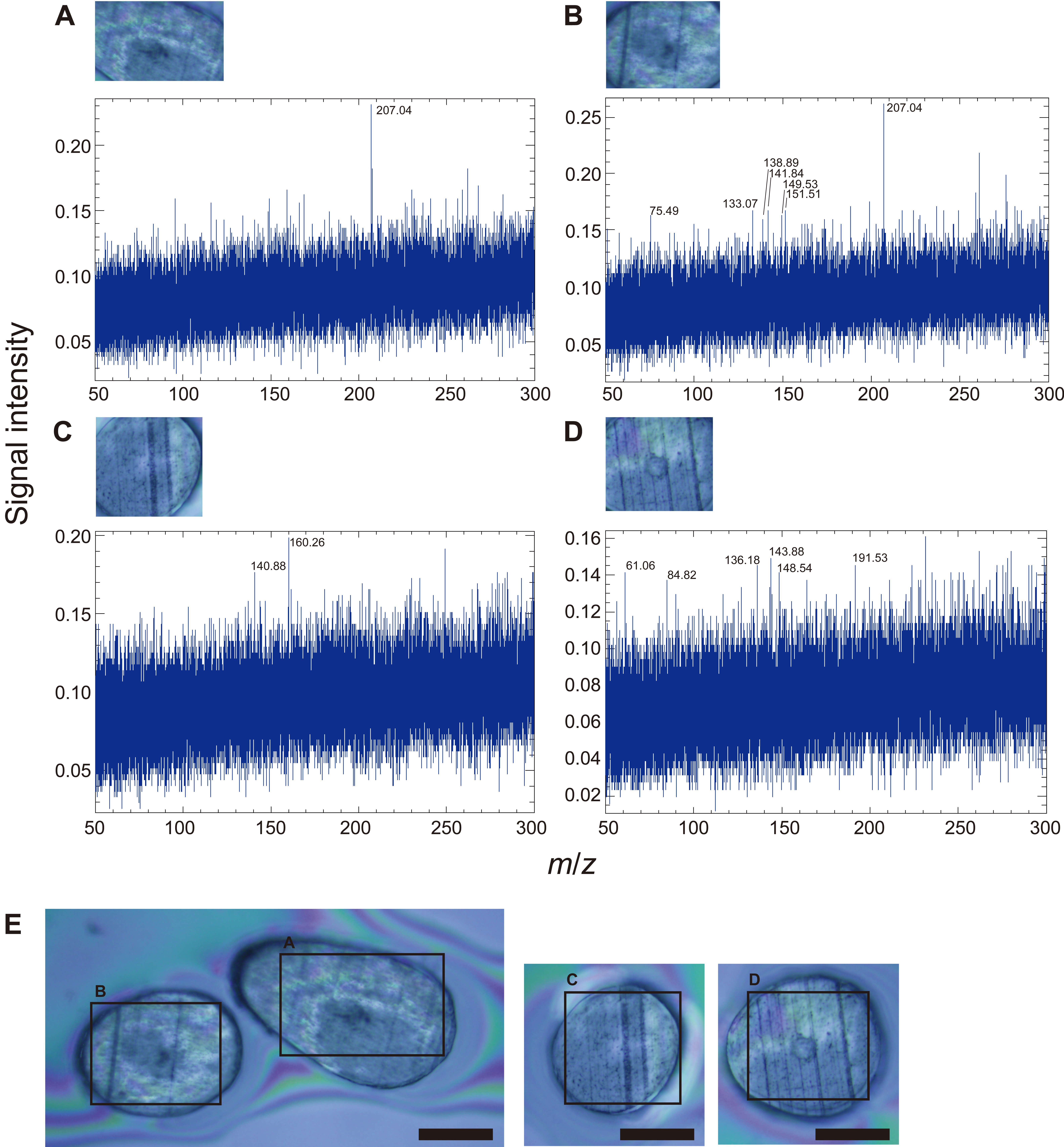

Supplement: Figure S4 — MS/MS analysis. (A–D) Mass spectra of secondary ions by fragmentation of the precursor ion at m/z 207.04 are presented. Measurement of precursor ions at energy level of 0. (B) Fragmentation at energy level of 10. (C) Fragmentation at energy level of 30. (D) Fragmentation at energy level of 50. The inserted pictures present the area on the hair sections on which the lasor was pulsed for ionization. (E) Light microscopic images of the hair sections are shown. Lasor-pulsed areas are framed. Scale bar: 50 µm. (TIF) [file pone.0026721.s004.tif]
